# The Running Track of Government Responsibility During the Pandemic, Characteristic Analysis of Policy Documents Relevant to the COVID-19 Pandemic Released by Beijing Municipal Government in 2020

**DOI:** 10.3389/fpubh.2021.713879

**Published:** 2021-11-10

**Authors:** Ayan Mao, Yujie Yang, Wuqi Qiu

**Affiliations:** Division of Public Health Information, Institute of Medical Information and Library, Chinese Academy of Medical Sciences and Peking Union Medical College, Beijing, China

**Keywords:** descriptive analysis, policy document analysis, COVID-19 pandemic, Beijing (China), 2020

## Abstract

**Objective:** Provide a reference point for the division of labor during the collaboration of multiple departments and the planning for the prevention and control of the Covid-19 epidemic of departments of the Beijing Municipal Government, from the perspective of policy documents.

**Methods:** Policy documents and daily updates on COVID-19 cases published in 2020 are taken from the official website of Beijing Municipal Government and Beijing Municipal Health Commission. The characteristics of the pandemic situation and the content of relevant documents issued by different departments are described in five stages.

**Results:** There were 988 confirmed cases of COVID-19 in Beijing in 2020, and policy analysis covered 444 documents (257 policy documents and 187 explanations of policy). A total of 153 policy documents were directly issued by the Beijing Municipal Government and its 45 subordinate commissions and bureaus, while others were policy forwarding from the central government and its relevant departments, county-level governments of Beijing and other organizations. Most cases and documents emerged during the initial stage of the pandemic (Level-I of the Emergency Response, which is the most serious). It was found that as many as 109 documents published by Beijing Municipal Government during the Level I emergency response period were relevant to economic and social development, 83 documents were related to disease control and medical services, and the rest were in close relation to the production and daily life of the people. Overall, major policy measures taken were relevant to 7 fields: finance, transportation, economic activities, employment people's lives, epidemic prevention and control and medical insurance. Policy implementation objectives were centered on promoting epidemic prevention and control and maintaining the stability of social production and residents' life. However, there are different emphases in different stages of the epidemic.

**Conclusion:** Beijing municipality realized an effective mode of collaboration among multiple departments and organizations in the prevention and control of the COVID-19 epidemic, which was an example of the practice of “Health in All Policies.”

## Background Information

Prevention of and response to a public health emergency involves effective integration and comprehensive utilization of different resources and capabilities of multiple departments and social groupings (1). Indeed, the cooperation of multiple departments is one of the core elements of modern public health theory and practice, playing an important role in the response to public health emergencies (2–5). In the research published so far, more attention has been paid to *health* policies relevant to epidemic prevention and control rather than relevant policies issued by other fields and their role in epidemic prevention and control (6). Some studies have shown that the level to a country's early policy attention to COVID-19 is highly related to its epidemic control effect. Therefore, it is necessary to systematically sort out the epidemic prevention and control policies in order to reveal the ways and modes in which these policies play a role (7).

Starting from analysis of the policy documents published by the Beijing Municipal Government in the special column “Beijing Fights against COVID-19” of the municipal website in 2020, this paper describes the ways that different governmental departments participated in the prevention and control of the epidemic and their priorities. It aims to provide a reference point for the division of labor during the collaboration and planning for epidemic prevention and control of multiple departments through a descriptive analysis of the roles of different departments of Beijing Municipal Government and their mode of cooperation during the work of prevention and control.

The prevention and control of the Covid-19 epidemic in Beijing Municipality withstood the test of three waves of impact, from the imported cases from Wuhan in January 2020, the imported cases from abroad in late February to the cluster of cases in Xinfadi Market (major agricultural products and seafood wholesale market in Beijing) on June 11. Although the city confronted the greatest variety of COVID-19 cases and a very long duration of restrictions, the measures it took were effective and progress was visible. On September 25, 2020, the Beijing Municipal Government reviewed and approved the *Regulations of Beijing Municipality on Public Health Emergency Response*, which summarizes the experience of the city in epidemic prevention and control and provides standard guidance for future work. The *Regulations* highlights the responsibilities of four parties, namely the local government, regulatory authorities, non-government organizations and individuals within the Beijing municipal jurisdiction. It is the first time that such local regulations included multiple departments' cooperation in public health emergency control systems in China. The regulatory authorities included transportation, education, commerce, grain, strategic reserves, emergency response, drug administration, development and reform, state property management, market regulation, health, ecology and environment, urban management, public security, civil affairs, medical security, agriculture and rural affairs, press and publicity, cybersecurity and information, science and technology, and foreign affairs departments, as well as other departments relevant to the urban administration.

## Research Methods

### Data Source

Documents: The policy documents were collected from the policies section of the special column “Beijing Fights against COVID-19” on the official website of Beijing Municipal Government (http://www.beijing.gov.cn/ywdt/zwzt/yqfk/). A total of 466 documents were collected, covering the period from January to December 2020.

The pandemic data: Data for the number of cases of Covid-19 was taken from the “Daily Update on COVID-19 Cases in Beijing” as published in the column “COVID-19 Pandemic Prevention and Control” on the official website of Beijing Municipal Health Commission (http://wjw.beijing.gov.cn/wjwh/ztzl/xxgzbd/). The daily update on COVID-19 cases in Beijing has been published since January 20, 2020. The data for this study were collected from January 20 to December 31 in 2020.

### Data Processing

Documents: 22 documents irrelevant to the study (including diagrams, posters, circulars on commendation and others) were excluded, so a total of 444 policy documents were included in the final analysis. A database for statistics was established with these documents. There are seven core variables: title, publication date, nature (policy and explanation of policy), publication organization, joint publication organizations, major contents and field. The topic/ field assignment to policy was completed independently by two members of the project team. When there was a discrepancy between their results, the final decision was made by the project team through group discussion.

Data of the Epidemic: the number of new cases was counted on a daily basis and further divided into the number of local confirmed cases and the number of imported confirmed cases. Suspected cases and asymptomatic cases were not included.

### Statistical Analysis

We perform the correlation analysis between the daily number of confirmed cases and the time distribution of documents published by governmental departments. For this purpose, the work of emergency response in 2020 was divided into five stages according to the level of response activated by the Beijing Municipal Government, namely Level I emergency response (January 24–April 30), the first Level II emergency response (May 1–June 5), the first Level III emergency response (June 6–June 15), the second Level II emergency response (June 16–July 19), and the second Level III emergency response (July 20–December 31). The characteristics of the development of the epidemic and the time distribution and content of relevant documents issued by different departments are described for each of the stages.

## Results

### Description of the Characteristics of the Unfold of the Epidemic at Different Stages

In total during 2020, there were 988 confirmed COVID-19 cases in Beijing, of which 986 were confirmed between January 20 and December 31 and 2 before January 20. During the stage of Level I emergency response (January 24 to April 30), the accumulated number of confirmed cases was 573, including 401 local cases (69.98%) and 172 imported cases (30.02%). It should be noted that there were two peaks in the number of confirmed cases at this stage. Most confirmed cases were local before March 5 while the number of imported cases increased remarkably afterward during the other peak. At the stage of the first Level II emergency response (May 1–June 5), there were no confirmed cases. The stage of the first Level III emergency response (June 6–June 15) marked the beginning the outbreak in Xinfadi Market in which all 106 confirmed cases were local. The stage of the second Level II emergency response (June 16 to July 19) covered the spreading and ending of the outbreak in Xinfadi Market, in which all 229 confirmed cases were local. At the stage of the second Level III emergency response (July 20 to December 31), there were 58 confirmed cases, including 29 local cases (50.00%) and 29 imported cases (50.00%).

**Figure 1:**
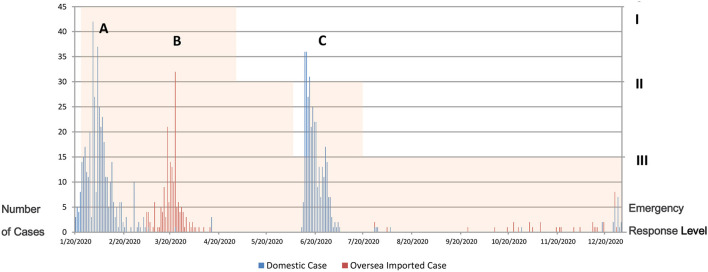
Daily confirmed COVID-19 cases in Beijing from January 20 to December 31, 2020. Peak A (January 20—February 20) was the outbreak of local cases during the Level I emergency response; Peak B (March 5—April 14) was the increase of imported cases during the Level I emergency response; Peak C (June 11—July 5) was the outbreak of local cases at Xinfadi Market from the first Level III emergency response to the second Level II emergency response.

Based on this distribution, three peaks of the confirmed cases in Beijing were noted. Peak A (January 20–February 20) was the outbreak of local cases during the Level I emergency response; Peak B (March 5–April 14) was the increase of imported cases during the Level I emergency response; Peak C (June 11–July 5) was the outbreak of local cases at Xinfadi Market from the first Level III emergency response to the second Level II emergency response (Figure 1).

### Description of the Attribute of Documents

Among all the documents we examined, 257 (57.88% of the total number) are policies and 187(42.127%)are explanations of policies. Of the 257 policies, 153 were published by Beijing Municipal Government and its subordinate 45 commissions and bureaus on the official website of the Beijing Municipal Government, 8 were published by county-level governments of Beijing and 91 were policy forwarding from the central government and its relevant departments. The 187 policy explanations documents were made by 46 departments and organizations, which were mostly published within 1 or 2 days after the release of policies by the relevant government departments (118 documents) or of reports by news and internet media (69 documents). As for the time distribution, 394 (88.74%) were published at the stage of the Level I emergency response, 2 (0.45%) at the stage of the first Level II emergency response, 1 (0.23%) at the stage of the first Level III emergency response, 31 (6.98%) at the stage of the second Level II emergency response, and 16 (3.60%) at the stage of the second Level III emergency response. The topics of the documents varied: 203 (45.72%) focus on the economy and social development, 127 (28.60%) were about disease prevention and control, and 77 (17.34%) were around people's daily life (Table 1).

**Table 1:** Features of policy documents relevant to the COVID-19 pandemic released by Beijing municipal government in 2020.

**Category**	**Number (*n*)**	**Proportion** **(%, *n*/444)**
Type (*N* = 444)		
Policy	257	57.88
*Beijing Municipal Government and its subordinate commissions and bureaus*	153	34.46
*Beijing county-level governments*	8	1.80
*Central Government*	91	20.50
*Other organizations*	5	1.12
Explanation of policy	187	42.12
*The relevant government departments*	118	26.58
*News and internet media*	69	15.54
Time distribution (*N* = 444)		
Level I emergency response	394	88.74
First Level II emergency response	2	0.45
First Level III emergency response	1	0.23
Second Level II emergency response	31	6.98
Second Level III emergency response	16	3.60
Content^*^ (*N* = 516)		
People's daily life	77	17.34
Health care	10	2.25
Disease prevention and control	127	28.60
Health insurance & assistance	21	4.73
Economy and social development	203	45.72
Community service	8	1.80
School related service	15	3.38
Employment and human resource	30	6.76
Transportation	21	4.73
Laws and regulations	4	0.90

* *Some documents involve more than one area of content classification, so N = 516 for content is greater than the total number of documents*.

### Distribution of Publishing Authorities

A total of 153 policy documents were published by Beijing Municipal Government and its subordinate 45 commissions and bureaus on the official website of the Beijing Municipal Government during the COVID-19 pandemic. The top five publishing authorities are the Beijing Municipal Human Resources and Social Security Bureau (19 documents), the Beijing Municipal Government (15 documents), the Beijing Municipal Commission of Housing and Urban-Rural Development (14 documents), the Beijing Municipal Commerce Bureau (10 documents), and the Beijing Municipal Bureau of Finance (6 documents).

As for the distribution in terms of the stage of emergency response, 128 documents were published at the stage of Level I emergency response and most focused on the Social development and security related fields (36), Economic related fields (27) and Comprehensive field (21). No documents were published at the stage of the first Level II emergency response. Only one document was published (by the Beijing Municipal Comprehensive Law-enforcement Bureau) at the stage of the first Level III emergency response. Fourteen documents were published at the stage of the second Level II emergency response and most focused on Social development and security related fields (10). Ten documents were published at the stage of the second Level III emergency response and most focused on Social development and security-related fields (4) and Education and cultural cultivation related fields (2) (Table 2).

**Table 2:** Information on policy documents issued by the Beijing municipal government and related departments on the official website during the COVID-19 in 2020.

**Authorities**	**Level I**	**First Level II**	**First Level III**	**Second Level II**	**Second Level III**	**Total**
**Comprehensive field**						
**Total**	**21**	**0**	**0**	**0**	**0**	**21**
*Beijing municipal government*	15	0	0	0	0	15
*Beijing municipal committee of the communist party of China*	5	0	0	0	0	5
*Standing committee of Beijing municipal people's congress*	*1*	0	0	0	0	1
**Social development and security related fields**						
**Total**	**36**	**0**	**0**	**10**	**4**	**50**
*Beijing development and reform commission*	3	0	0	1	1	5
*Beijing municipal commission of housing and urban-rural development*	11	0	0	4	0	15
*office of beijing housing provident fund management committee*	0	0	0	1	0	1
*beijing municipal bureau of human resources and social security*	15	0	0	2	3	20
*beijing social insurance fund management center*	3	0	0	1	0	4
*beijing municipal health insurance bureau*	4	0	0	1	0	5
**Economic related fields**						
**Total**	**27**	**0**	**0**	**0**	**2**	**29**
*Beijing municipal commerce Bureau*	8	0	0	0	2	10
*Beijing municipal bureau of finance*	6	0	0	0	0	6
*Beijing municipal Bureau of market supervision*	5	0	0	0	0	5
*Beijing municipal bureau of local financial supervision*	2	0	0	0	0	2
*Beijing municipal bureau of economy and information technology*	2	0	0	0	0	2
*Beijing federation of trade unions*	2	0	0	0	0	2
*Management committee of Beijing economic and technological development zone*	1	0	0	0	0	1
*Beijing regulatory Bureau of China bank insurance regulatory commission*	1	0	0	0	0	1
**Education and cultural cultivation related fields**						
**Total**	**14**	**0**	**0**	**1**	**3**	**18**
*Beijing municipal commission of education*	3	0	0	1	0	4
*Beijing Municipal Bureau of sports*	3	0	0	0	1	4
*Beijing municipal Bureau of culture and tourism*	3	0	0	0	1	4
*Office of beijing leading group for cultural reform and development*	2	0	0	0	0	2
*Beijing film Bureau*	0	0	0	0	1	1
*Beijing park management center*	1	0	0	0	0	1
*Beijing state owned cultural assets management center*	1	0	0	0	0	1
*Beijing municipal Bureau of cultural relics*	1	0	0	0	0	1
**Livelihood and resource related fields**						
**Total**	**15**	**0**	**0**	**2**	**1**	**18**
*Beijing municipal commission of planning and natural resources*	3	0	0	0	0	3
*Beijing municipal Bureau of water resources*	3	0	0	0	0	3
*Beijing transportation commission*	2	0	0	0	0	2
*Beijing municipal commission of science and technology*	2	0	0	1	0	3
*Beijing municipal bureau of civil affairs*	1	0	0	1	1	3
*Beijing housing provident fund management center*	2	0	0	0	0	2
*Beijing municipal bureau of ecological environment*	1	0	0	0	0	1
*Office of the special class for the rectification of illegal group rental housing in Beijing*	1	0	0	0	0	1
**Public health and medical related fields**						
**Total**	**4**	**0**	**0**	**1**	**0**	**5**
*Beijing municipal commission of health*	1	0	0	1	0	2
*Beijing New coronavirus pneumonia epidemic prevention and control work leading group office*	2	0	0	0	0	2
*Beijing drug administration*	1	0	0	0	0	1
**Legal and regulatory related fields**						
**Total**	**6**	**0**	**0**	**0**	**0**	**6**
Beijing municipal Bureau of justice	1	0	0	0	0	1
*Beijing municipal commission of urban administration*	1	0	0	0	0	1
*Beijing municipal commission for discipline inspection*	1	0	0	0	0	1
Beijing emergency management Bureau	1	0	0	0	0	1
Beijing civil air defense office	1	0	0	0	0	1
Beijing customs of the People's Republic of China	1	0	0	0	0	1
**Total**	**128**	**0**	**1**	**14**	**10**	**153**

### Distribution of Contents of Policy Documents in Different Periods

**Figure 2:**
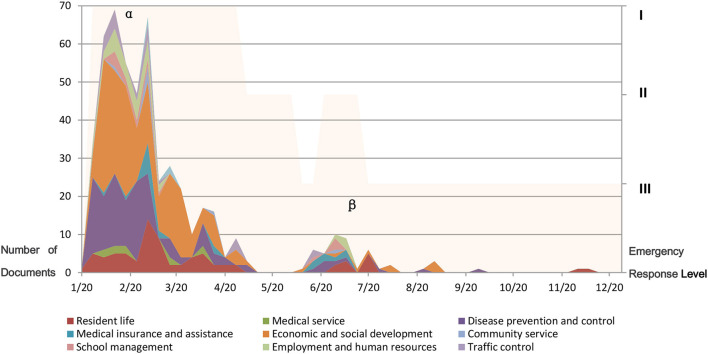
Classification of Daily content of policy documents on the official website of the Beijing Municipal Government from January 20 to December 31, 2020. Peak α was the peak of policy documents publishing during the stage of the Level I emergency response; Peak β was the peak of policy documents publishing during the stage of the second Level II emergency response.

Considering the time distribution, there were two peaks for the publishing of policy documents. The higher peak (Peak α) was during the stage of the Level I emergency response and the other peak (Peak β) was during the stage of the second Level II emergency response (Figure 2). Considering the contents of the policy documents, most of them were about economic and social development (116 documents), followed by disease prevention and control (76 documents) and people's daily life (43 documents). The majority of these documents were published during the stage of Level I emergency response and all fields were covered. It should be noted that although the total number of documents published was small during the stage of the second Level II emergency response, the contents also involved severe aspects, namely people's daily life, disease control, medical insurance and assistance, economic and social development, school management, employment and human resources, and transportation management (Table 3).

**Table 3:** Contents of policy documents of Beijing municipal government website during the stages of the 2020 COVID-19 epidemic.

**Content**	**I**	**II**	**III**	**II-2**	**III-2**	**Total**
People's daily life	37	0	0	2	4	43
Health care	5	0	0	0	0	5
Disease prevention and control	70	0	0	4	2	76
Health insurance & assistance	8	0	0	3	0	11
Economy and social development	109	0	1	2	4	116
Community service	5	0	0	0	0	5
School related service	6	0	0	2	0	8
Employment and human resources	14	0	0	2	0	16
Transportation	10	0	0	1	0	11
Laws and regulations	3	0	0	0	0	3
**Total**	**267**	**0**	**1**	**16**	**10**	**294**

### Distribution of Key Information in Policy Documents Published During the Three Peaks of Confirmed Cases

In this analysis, emphasis is laid on the analysis of key information of the policy documents published during the three peaks of confirmed cases, including the major targets, measures, and purpose of the policies. To be specific, this study analyzed the targets of the policies, the major measures taken, and the purposes of these major measures.

The targets of the policies can be divided into two categories, namely organizations (venues) and groups of people. The targets of the relevant policies differed at the three peaks of confirmed cases. During Peak A, the policy documents pay more attention to public sites, institutions, industries and the general population. During Peak B, the focus of attention was further extended to local enterprises and people relevant to the prevention and control of COVID-19 pandemic (such as frontline medical staff, discharged patients, foreign patients). During Peak C, the target of policy attention has shrunk sharply with narrower coverage. Generally speaking, due to the continuity of the policies, Peak A belongs to the foundation laying period, with large and comprehensive policy coverage; Peak B belongs to the policy adjustment and improvement period, according to the progress of the epidemic, new policy targets have been included in succession; Peak C is a period of policy stability, with some minor adjustments (Table 4).

**Table 4:** Key points of policy documents issued in municipal government website during three outbreak peaks of the 2020 COVID-19 epidemic in Beijing.

**Key points**		**Peak-A**	**Peak-B**	**Peak-C**
Target	Organizations: Sites, industries and institutions	**Sites (8):** villages, communities, residential areas, offices, public places, construction sites, shopping malls, business buildings; **Enterprises (5):** insured enterprises, key guarantee enterprises, enterprises with business difficulties, medium-, small- and micro-sized enterprises, property service enterprises; **Industries (11):** catering industry, service industry, retail industry, foreign trade enterprises, foreign-funded enterprises, cultural industry, breeding industry, medical and health industry, pharmaceutical industry, transportation service industry, leasing intermediary industry; **Institutions (5):** schools, pension institutions, medical institutions, travel agencies, land trading offices;	**Sites (6):** pedestrian streets, large shopping malls, construction sites, tourist attractions, business buildings, key sites; **Enterprises (7):** service trade, high precision industries, industrial enterprises, private enterprises, domestic and foreign trade enterprises, foreign trade enterprises, sports enterprises, medium-, small- and micro-sized enterprises; **Industries (6):** transportation, transportation services, tourism, civil aviation, pig production related industries, housing rental services; **Institutions (8):** colleges and universities, gas power plants, thermal power plants, market supervision departments, enterprises and institutions, pension service institutions, medical institutions, key institutions;	**Sites (2):** Construction sites, residential areas; **Industry (2):** transportation, education; **Institution (1):** pension service institutions;
	People	**General population (8):** college graduates, family members, labor force, migrant workers, employees, the public, underage children, key groups. **COVID-19 related population (1):** frontline medical staff;	**General population (10):** college graduates, self-employed people, the public, children without guardianship, people in need, the elderly, migrant workers, people with special difficulties, key groups, overseas returnees; **COVID-19 related population (5):** frontline medical staff, discharged patients, foreign patients, dead patients, martyrs; **Wuhan epidemic related personnel (2):** people in and out of Hubei Province, Beijing citizens staying in Hubei province;	**General population (1):** college graduates;
Mechanism		**Finance related (6)**: loans, financial services, special fund support, financing guarantee, interest discount, service subsidies; **Transportation (5)**: vehicle tolls, in and out Beijing management, spring return peak, toll roads, emergency transport vehicles; **Economic activities (11)**: enterprise development, entrepreneurship, short-term export, listing guidance, bidding, credit insurance, liability insurance, social insurance, production license, registration and approval; **Employment (4)**: wages, employment security, labor relations, personal evaluation; **Residents' life (8)**: air conditioning and ventilation system, food supply, agricultural products, group dinners, online classrooms, delayed school start, cloud office, service vouchers; **Epidemic prevention and control (7)**: segregation, inspection and law enforcement, hierarchical and divisional prevention and control, closed management, emergency treatment, emergency approval, major responsibility system **Medical and insurance (6)**: basic medical needs, COVID-19 diagnosis and treatment scheme, medical services, medical protective clothing, medical preparations, medical security;	**Finance related (6)**: real estate, guaranteed loans, entrepreneurship guaranteed loans, development funds, capital subsidies; **Economic activities (4)**: entrepreneurship, gas for commercial use, basic endowment insurance, social insurance premium, unemployment insurance; **Employment (3)**: recruitment, employment, labor relations; **Residents' life (8)**: spring semester, middle and primary school curriculum, afforestation in spring, point-to-point travel, cross-provincial cross-border tourism, cultural and sports activities, retail prices; **Epidemic prevention and control (6)**: frontier health quarantine, wearing masks, epidemic prevention and control strategy, normalization and precision of epidemic prevention and control, community prevention and control, rescue and protection **Medical and insurance (8)**: COVID-19 diagnosis, treatment capacity, re-visit and re-examination, health services, health management, vaccination, medical expenses payment, industrial injury insurance premium;	**Economic activities (1)**: Enterprise social insurance premiums **Residents' life (3)**: electricity cost, housing accumulation fund, interior decoration; **Epidemic prevention and control (2)**: emergency response, secondary level epidemic response; **Medical and insurance (3)**: COVID-19 diagnosis, COVID-19 detection capability, outpatient prescription service

Major measures taken were relevant to 7 fields: **① Finance** measures were mostly about loans, development funds, subsidies, and so on; **② Transportation** measures were concentrated during Peak A and mainly concerned the control of the entry to and exit from Beijing and emergency transportation; **③ Economic activities** measures included insurance and support for entrepreneurship; **④ Employment** measures addressed labor relations, salary levels, and employment; **⑤ People's lives** measures covered a wide range from the food supply, education, working online, water and power supply to culture and tourism; **⑥ Epidemic prevention and control** measures include quarantine, protection for frontline health workers, classified prevention and control, and normalized epidemic prevention and control; **⑦ Medical insurance** measures were mainly about the payment for virus testing as well as treatment of COVID-19 patients and payment of expenses (Table 4).

Policy implementation objectives were centered on the objective of promoting epidemic prevention and control and maintaining the stability of social production and residents' life, though at different peak periods with varying attention. Peak A highlighted the promotion of consumption, resumption of work and production, and social stability. Peak B highlighted the promotion of sound and stable development of the economy especially the stability in production, foreign trade, foreign investment, and employment. During Peak C two aims were highlighted: to guard against rebounding of the epidemic and normalize epidemic prevention and control; to promote resumption of work and production.

## Discussion

### Putting the Strategy of “Health in All Policies” Into Practice in the Prevention and Control of the COVID-19 Pandemic by Beijing Municipality

The health of people is affected by the policies formulated by not only the health department but also other government departments such as education, agriculture, and the environment. Meanwhile, the promotion and implementation of health-related policies also involves all walks of life and requires supporting policies in other sectors. “Health in All Policies” (HiAP) proposed by the WHO is an approach to public policies across sectors that systematically takes into account the health implications of decisions, seeks synergies, and avoids harmful health impacts to improve population health and health equity (8, 9). Looking back on the development of public health in China, “Health in All Policies” has been incorporated into the governance on a range of public health activities from “patriotic sanitation campaigns” to the construction of “patriotic healthy cities” and “healthy cities” as a basic strategy for promoting the sustainable development of population health (10, 11).

Beijing Municipality has put the strategy of “Health in all Policies” into its policies for epidemic prevention and control. Judging from the analysis of the departments and organizations publishing policy documents, epidemic prevention and control is probably the health work most widely adopted by all government departments and organizations. There are in total 53 departments and organizations under Beijing Municipal Government (including 26 departments, 1 special organization, 11 direct affiliates, and 15 other organizations). It was found that 45 of them published policy documents on related work during the epidemic, so nearly 85% of the governmental departments and organizations participated in the epidemic prevention and control in their ways. Covering a wide range of topics such as daily life, medical service, disease control, medical security, social assistance, and economic life and social construction, these policy documents included both the supporting policies for the implementation of measures for prevention and control of the epidemic in corresponding industries and fields and the adjustment of relevant policies in order to give full play to their supporting role in epidemic prevention and control of the whole city. The rights and obligations of different departments in the epidemic prevention and control are clarified in the form of “the responsibilities of four parties (the local government, regulatory authorities, non-government organizations and individuals)” in the *Regulations of Beijing Municipality on Public Health Emergency Response* issued by Beijing Municipality in September 2020, which not only systematically summarizes the experience in previous work but also incorporates the concept of “Health in All Policies” into the work system for epidemic prevention and control through legislation.

### Relationship Between the Publishing Time and Priorities of Policy Documents

The first peak (Peak α) of the publishing of policy documents basically covered the first two peaks of the number of confirmed cases (Peak A and Peak B) during the Level I emergency response in Beijing. Accounting for 90% of the policy documents published, these documents involved a wide range of fields and work modes. The COVID-19 pandemic caused huge social and economic losses. The rationale was that if the social and economic stability could not be maintained, it would, in turn, weaken the epidemic prevention and control and thus form a vicious circle (12–14). Therefore, both the economic and social stability and the policies for prevention and control of the epidemic were important for controlling the spread of the epidemic and safeguarding the people's health. It was found that as many as 109 documents published by Beijing Municipal Government during the Level I emergency response period were relevant to economic and social development, 83 documents were related to disease control and medical services, and the rest were in close relation to the production and daily life of the people, such as management of the community, schools, employment, and transportation. It can be said that it is the coordination of various policies of different sectors that promoted the work of epidemic prevention and control in Beijing. Indeed, the epidemic was effectively controlled.

The second peak of the publication of policy documents appeared during the second Level II emergency response, which is closely related to the outbreak of local cases at Xinfadi Market. Considering that many previously released policies were still in effect, the policies published in the period aimed to supplement and enhance those policies to address the new situation. More attention was paid to the arrangements for coping with the prevention and control of the local outbreak. Half of the documents were about epidemic prevention and control and medical security, while new regulations were also made for the management of schools and students, people's daily life, and transportation.

### Measures and Objectives of Policy Documents Varies at Different Stages of the Epidemic

Policies for safeguarding the health of the people supported the whole process of the all-around arrangements for epidemic prevention and control and medical treatment. Such documents account for over 30% of the total number of documents published. During the Peak A period, most of the confirmed cases in Beijing were local. In the beginning, different parties went their own way in epidemic prevention and control, yet the work was gradually standardized through joint efforts of relevant departments. Through emphasizing such measures as emergency response, quarantine, strict supervision and law enforcement, classified prevention and control, closed-loop control, and the allocation of major responsibilities, the work mode and management standards for prevention and control of the epidemic in Beijing were established. In terms of medical services, the testing and treatment of COVID-19 were standardized while other basic medical needs of the people were protected. During the Peak B period, although most of the confirmed cases were imported, there were occasional confirmed local cases. Attention was paid to the arrangements for the protection of frontline workers, rescue and emergency support, standardization of the strategy for prevention and control of the epidemic and its implementation, community work in preventing and controlling the epidemic, and normalizing the strategies for prevention and control of the epidemic. In terms of medical treatment, the effectiveness of virus detection and clinical treatment, as well as the efficacy of vaccine, was highlighted. During the Peak C period, there was a local outbreak. Because there was basically a sound system for prevention and control of the epidemic in place, attention was paid to the rights and responsibilities of different sectors in emergency response while the measures for Level II emergency response were standardized. For medical services, large-scale virus detection was highlighted to serve for the screening of infected groups.

More attention was paid to social stability, the normal order for life and work, and a sufficient supply of epidemic prevention materials and daily necessities during Peak A and B. As for people's daily life, it was stressed that all people needed to reduce gatherings and dining together and refrain from traveling, while schools were required to postpone the reopening and carry out online teaching to avoid the spread of the infection. During Peak B in particular, the entry-exit controls was enhanced to guard against COVID-19 cases from abroad. For employment, the level of the minimum wage and employment security were stressed to maintain stable employment. Meanwhile, working online was encouraged to alleviate the pressure on organizations and enterprises from epidemic prevention and control. For transportation, the control of the entry to and exit from Beijing was strengthened while green channels for ensuring the supply of necessities were opened. The policy system for stabilizing economic development was established. In the early stage, attention was paid to supporting micro, small and medium enterprises, by reducing or waiving tax and administrative fees, cutting interest rates, providing subsidies, and so on. Later, attention was shifted to the resumption of work and production to invigorate the economy. The policies of tax reduction and exemptions, and subsidies were further used to encourage business startups.

A study analyzed the policies released globally during the COVID-19 pandemic and found that in the early stage, about 90% of COVID-19 policy documents belong to the scientific and technical aspects of epidemic prevention, and in the middle and late stage, a rise in attention to social, economic development and human resource (7). This is generally consistent with our findings, but in the initial stage of the epidemic, policies related to social, economic development and human resources accounted for a much higher proportion in Beijing. This result may suggest that the policy release of the Beijing municipal government was more comprehensive and covers a wider range even from the initial stage.

### Potential Limitations and Future Works

Several problems and deficiencies may limit the effectiveness of this study. The source of documents was limited to the official website of Beijing Municipal Government, so it could not cover official documents within the government that are not open to the public. Access to more documents on the coordination and cooperation of municipal departments might usefully supplement these documents. Similarly, due to time and space constraints, the analysis was unable to cover the documents published by the central government, or by the districts and counties of Beijing. These should be added in any further research. Also, for instance, the paper does not mention the planning for prevention and control in multiple departments, or how they managed their collaboration, but only reflects the multi-department cooperation based on the description of the release of the policy documents, which has certain limitations.

## Conclusion

Beijing municipality realized a useful mode of collaboration by multiple departments and organizations in the work of prevention and control of the Covid-19 epidemic. As an example of the practice of “Health in All Policies,” this collaboration effectively supported the work of prevention and control of the Covid-19 epidemic and helped maintain the stable operation of society and the economy and the stability of people's life. Also, at different stages of the pandemic, the focus of policy attention and its mode of action and measures have played an important role in the effective control of the epidemic in Beijing. We believe that these comprehensive policy models of epidemic control of Beijing municipal government can provide experience for other countries and regions.

## Data Availability Statement

The datasets presented in this study can be found in online repositories. The names of the repository/repositories and accession number(s) can be found below: http://www.beijing.gov.cn/ywdt/zwzt/yqfk/.

## Author Contributions

AM is responsible for research design, data analysis, and thesis writing. YY participated in data analysis and thesis writing. WQ participated in project design and coordination of research activities. All authors contributed to the article and approved the submitted version.

## Funding

This work was supported by Peking Union Medical College Education Foundation Funded projects and Capital health development scientific research project (2021-1G-4251).

## Conflict of Interest

The authors declare that the research was conducted in the absence of any commercial or financial relationships that could be construed as a potential conflict of interest.

## Publisher's Note

All claims expressed in this article are solely those of the authors and do not necessarily represent those of their affiliated organizations, or those of the publisher, the editors and the reviewers. Any product that may be evaluated in this article, or claim that may be made by its manufacturer, is not guaranteed or endorsed by the publisher.
